# Multi-Functional Nanogels for Tumor Targeting and Redox-Sensitive Drug and siRNA Delivery

**DOI:** 10.3390/molecules21111594

**Published:** 2016-11-23

**Authors:** Giorgia Adamo, Natascia Grimaldi, Simona Campora, Donatella Bulone, Maria Luisa Bondì, Mohamad Al-Sheikhly, Maria Antonietta Sabatino, Clelia Dispenza, Giulio Ghersi

**Affiliations:** 1 Dipartimento di Scienze e Tecnologie Molecolari e Biomolecolari, Università degli Studi di Palermo, Viale delle Scienze, Edificio 16, 90128 Palermo, Italy; giorgia.adamo@unipa.it (G.A.); simona.campora@unipa.it (S.C.); 2 Dipartimento di Ingegneria Chimica, Gestionale, Informatica, Meccanica, Università degli Studi di Palermo, Viale delle Scienze, Edificio 6, 90128 Palermo, Italy; ngrimaldi@icmab.es (N.G.); mariaantonietta.sabatino@unipa.it (M.A.S.); clelia.dispenza@unipa.it (C.D.); 3 Consiglio Nazionale delle Ricerche (CNR)—Istituto di Biofisica (IBF) UOS Palermo, Via U. La Malfa 153, 90146 Palermo, Italy; donatella.bulone@pa.ibf.cnr.it; 4 Consiglio Nazionale delle Ricerche (CNR)—Istituto per lo Studio dei Materiali Nanostrutturati (ISMN) UOS Palermo, Via Ugo La Malfa, 153, 90146 Palermo, Italy; bondi@pa.ismn.cnr.it; 5 Department of Materials Science and Engineering, University of Maryland, College Park, MD 20742, USA; mohamad@umd.edu

**Keywords:** PVP, nanogels, e-beam, folate-targeting, doxorubicin, siRNA, GSH-responsive release

## Abstract

(1) Background: A new family of nanosystems able to discern between normal and tumor cells and to release a therapeutic agent in controlled way were synthetized by e-beam irradiation. This technique permits to obtain biocompatible, sterile, carboxyl-functionalized polyvinylpyrrolidone (PVP-co-acrylic acid) nanogels (NGs); (2) Methods: Here, we performed a targeting strategy based on the recognition of over-expressed proteins on tumor cells, like the folate receptor. The selective targeting was demonstrated by co-culture studies and flow cytometry analysis, using folate conjugated NGs. Moreover, nanoparticles were conjugated to a chemotherapeutic drug or to a pro-apoptotic siRNA through a glutathione sensitive spacer, in order to obtain a controlled release mechanism, specific for cancer cells. The drug efficiency was tested on tumor and healthy cells by flow cytometric analysis, confocal and epifluorescence microscopy and cytotoxicity assay; the siRNA effect was investigated by RNAi experiment; (3) Results: The data obtained showed that the use of NGs permits a faster cargo release in cancer cells, in response to high cytosolic glutathione level, also improving their efficacy; (4) Conclusion: The possibility of releasing biological molecules in a controlled way and to recognize a specific tumor target allows overcoming the typical limits of the classic cancer therapy.

## 1. Introduction

One of the most important goals in cancer therapy is the formulation of alternative systems for pharmacological treatments. Nanotechnologies have the potential to revolutionize the existing therapies, through innovative strategies able to deliver molecules to desired sites in the body, thus improving the therapeutic index of drugs. The use of nanoparticles (NPs) could increase the bioavailability of water-insoluble drugs allowing them to pass physiological barriers; moreover, they can carry large amount of cargo [1]. Tumor targeting approaches have been developed to enhance the effectiveness and reduce side effects by regulating the random cancer drugs biodistribution in the body [2].

It is well established that solid tumors are often characterized by the over-expression of specific molecules like antigens or receptors on their cell surface. Therefore, passive nanoparticles accumulation by the enhanced permeability and retention (EPR) effect can be associated with an active targeting, since nanoparticles can be functionalized with specific compounds able to link tumor cell surface molecules [3]. For instance, small molecules have received considerable attention as potential targeting ligands, due to their low molecular weight, ease of conjugation with nanoparticles and low production costs [4]. Folate, which is essential in many metabolic processes involved in cell survival, shows high specificity for its receptor that is over-expressed in many types of tumor cells. Indeed, it can be conjugated to NPs for a specific targeting in drug delivery systems [5]. For example, folate-functionalized liposomes were used for acute myelogenous leukemia treatment, while folate-conjugated methotrexate-loaded PAMAM(G5) dendrimer induced a tenfold reduction in cervical tumor size and exhibited less systemic toxicity, compared with free methotrexate [6,7]. Furthermore, nanocomplexes can be optimized in order to obtain a controlled drug release. Recently, various “smart nanosystems” have been developed to deliver drugs or biological molecules in response to an internal or external stimulus such as redox, temperature or pH variations: in this way it is possible to improve both in vitro and/or in vivo drug release profiles [4,8,9]. Indeed, the in depth knowledge of the target site (like tumor tissue) can be exploited to perform a specific drug controlled release nanosystem. For example, reduced glutathione (GSH) is well known to be implicated in the maintenance of cellular homeostasis in biological systems, acting as detoxifying agent toward reactive oxygen and xenobiotic species [10,11]. Indeed, GSH acts by inhibiting the toxic action of metabolites produced during normal metabolic processes or in stress condition that it typical of the tumor cells. The presence of a pendant thiol group (−SH) in GSH makes it a very efficient reducing agent present at different concentrations in intracellular and extracellular compartments [8]. It is well-known that the intracellular environment is different from extracellular in pH and redox states. In particular, intracellular glutathione concentration (~1–10 mM) is higher with respect to the extracellular GSH concentration (~2–20 μM). In addition, the cytosolic GSH level in cells with enhanced oxidative stress (like tumor cells) is 7–10 fold higher than normal cells [12]. In this view, a GSH-controlled release strategy is based on this largely difference of the redox potential between intracellular and extracellular compartments as well as the further elevated GSH concentration in tumor cells, compared to the healthy cells [9,10,12].

Herein, we have developed carboxyl functionalized poly(vinylpyrrolidone) (PVP) nanogels (NGs), produced by pulsed electron-beam irradiation. The choice of nanogels as substrate is motivated by the peculiar properties of these nanoparticles; their high colloidal stability when dispersed in aqueous media in a wide pH range and their shape change ability, provided that the crosslinking density is not too high. Their flexibility and shape changing ability can facilitate the bypass of biological barriers, ensure protection of the payload and enable interaction of the attached ligand with its receptors [13,14,15].

The synthetic methodology chosen for the production of nanogels utilizes ionizing radiation to induce inter- and intra-molecular crosslinking and functionalization of polymer chains in aqueous solutions. The distinctive advantage of this approach is that no surfactants are required to control the locus of reaction and that chemically stable polymers, such as PVP, can be used as a starting material. As a result, purification is simpler and products are non-toxic. Radiation doses within the sterilization dose range are applied to the systems.

The reactions constituting the basis for nanogel formation and the approaches to control particle size and functionality have been discussed in two recent reviews [16,17]. Briefly, N_2_O-saturated aqueous solutions of PVP are irradiated to short pulses of accelerated electrons. The ionizing irradiation interacts mainly with water producing the following species:

H_2_O → **^•^**OH, e^−^_aq_, H, H_2_, H_2_O_2_, H_3_O^+^

**Scheme 1:**
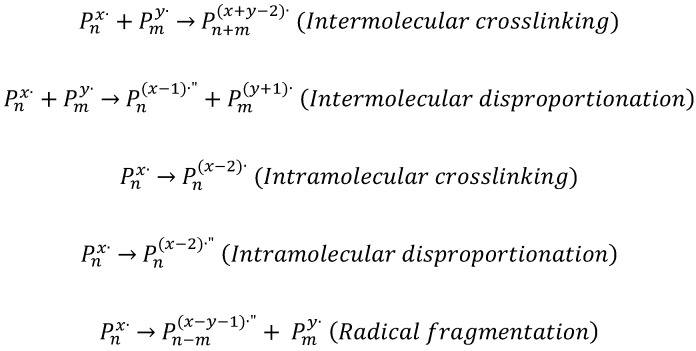
Possible reactions of polymer macroradicals. Apexes *x* and *y* are the number of radicals per polymer chain. Disproportionation and fragmentation results in the formation of double bonds (denoted with the apex ||).

In N_2_O-saturated aqueous solutions electrons are converted in hydroxyl radicals. **^•^**OH abstracts hydrogen atoms from the polymer forming macroradicals and can also recombine at low polymer concentration. The polymer carbon center radicals can undergo several possible reactions, depending on polymer concentration and dose rate, as reported in Scheme 1.

In the absence of oxygen, the main reactions of the polymer radicals are inter- and intra-molecular crosslinking. However, disproportionation reactions can also occur to less extent, leading to the formation of a double bond on one chain (when intermolecular) or on one site of the polymer chain (when intramolecular).

While the inter-molecular processes take place mainly between the lower molecular weight polymer molecules in the system, due to the lower number of free radicals per chain and their higher chain mobility, the intramolecular processes occur preferentially in the higher molecular weight chains, due to the higher number of radicals per chain and lower chain mobility. These two simultaneous processes lead to narrower molecular weight and particle size distributions, hence lower polydispersity values [18,19].

Under our experimental conditions, at high doses, molecular oxygen can be produced via the following reactions:

^•^OH + ^•^OH = H_2_O_2_

H_2_O_2_ + ^•^OH = H_2_O + HO_2_^•^

2 HO_2_^•^ = H_2_O_2_ + O_2_

and, in turn, react with the polymer radicals forming peroxyl radicals.

The peroxyl radicals can undergo further reactions, producing ketones and carboxyl groups. The most interesting chemical modification of PVP nanogels for the purpose of their application as biomedical nanocarriers is the formation of carboxyl groups and primary amino groups [18,19].

**Scheme 2:**
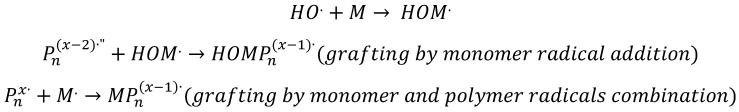
Possible reactions of radical bearing monomers with macroradicals or with double bonds formed on the polymer by disproportionation and fragmentation reactions.

The addition of a vinyl monomer to the polymer solutions introduces a competitive reaction between ^•^OH with the double bond, and ^•^OH with the polymer. This decreases the concentration of formed macroradicals. It is also expected that the monomer free radicals would react with the disproportionally-produced double bonds in the backbone of the polymer chain through addition reaction (grafting). The grafting of the monomer onto the polymer can also take place through the combination reactions of ${\mathrm{HOM}}^{.}$ with polymer radicals (see Scheme 2).

Since grafting competes with inter-molecular crosslinking reactions, it leads to comparatively smaller particles [20].

By varying the initial concentrations of polymer and monomer, dose-rate and total absorbed dose, one can enhance the probability of certain reactions taking place over others. As a result, PVP-co-AA nanogels with various molecular weights, hydrodynamic diameters and average concentration of functional groups per nanoparticle can be produced.

In particular, four PVP-co-AA systems were synthetized by varying the polymer and acrylic acid concentrations, and characterized for their hydrodynamic size, molecular weight, surface charge density and concentration of functional groups (carboxyl groups and primary amino groups). 

The available primary amino groups of the nanoparticles were covalently bonded to the carboxyl group of folic acid. The ability of the folate-conjugated nanoparticles to recognize tumor cells was tested using Hela cells (characterized by high expression of folate receptor) and NIH3T3 cells (a fibroblastic cells line used as negative control). 

Additionally, a selected nanogel system was conjugated to Doxorubicin or Bcl-2 siRNA through a redox-sensitive spacer in order to develop GSH-responsive nanosystems. The resulting nanosystems were characterized in vitro, measuring the drug release kinetics under reducing conditions. Moreover, their effects were evaluated both in tumor and healthy cells. The results show the DOX release from nanogels, triggered by higher intracellular GSH concentration, which resulted in enhanced inhibition of the cellular proliferation after internalization. Proof-of-principle experiment has also demonstrated the gene silencing ability of siRNA-loaded-nanogels on Hela cells under reducing conditions, providing evidence of their potential as nanomedicine carrier for the treatment of cancer.

## 2. Results

### 2.1. Nanogels Characterization and Decoration with Amino Fluorescein and Folic Acid

Polymeric crosslinked nanoparticles were produced by e-beam pulsed irradiation of PVP aqueous solutions in the presence of acrylic acid (AA) as described in a previous work [20]. Variants with different size and degree of functionalization were obtained from PVP aqueous solutions with polymer concentration in the 0.1 wt %–0.5 wt % range, adding AA at two molar ratios with respect to PVP repeating unit. The hydrodynamic diameters and weight average molecular weight, determined by dynamic laser scattering measurements and Zimm plot analysis, respectively, are shown in Table 1. The average number of carboxyl groups per nanoparticle was estimated via a colorimetric method that is based on Nickel complexation by carboxyl groups; primary amino groups were titrated upon reaction with fluorescamine. A detailed description of the protocols is reported elsewhere [19]. Results are presented in Table 2. Surface charge densities measured by Laser Doppler Velocimetry (LDV) are also reported in Table 2.

**Figure 1:**
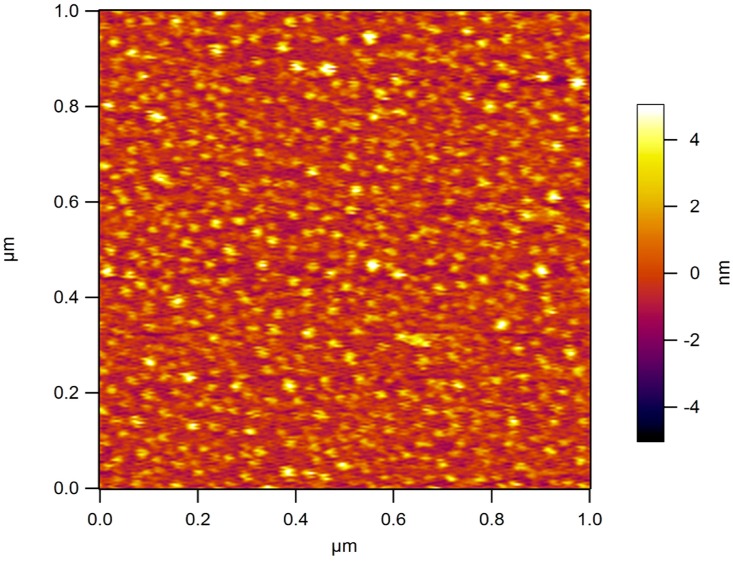
Height profile from tapping mode AFM under water for P*(0.5)AA50. The color scale represents a maximum height of 8 nm.

NGs with average hydrodynamic diameters in the 15–60 nm range with PDI < 0.3 were obtained. Both particle size and mass average molecular weight increase at the increase of polymer concentration, while are little affected by the increase of acrylic acid content for the same polymer concentration (0.25 wt %). P*0.1AA50 has the highest density of COOH groups while P*0.5AA50 has a significant concentration of both functional growth zeta potential values of about (−20)/(−50) mV. This is due both to the formation of carboxyl groups on PVP bydiation [19] and the acrylic acid grafted. P*0.25AA25 shows the highest value of zeta potential. The higher amount of AA with respect to P*0.25AA50 does not have any influence on the hydrodynamic size and molecular weight of the system, but strongly affects the surface charge density making these NGs more anionic as expected. A globular, flexible structure is supported by tapping mode atomic force microscopy in water. AFM images for P*(0.5)AA50 system are presented in Figure 1. Attractive interactions with the solid substrate induce the round-shaped nanogels to flatten.

**Figure 2:**
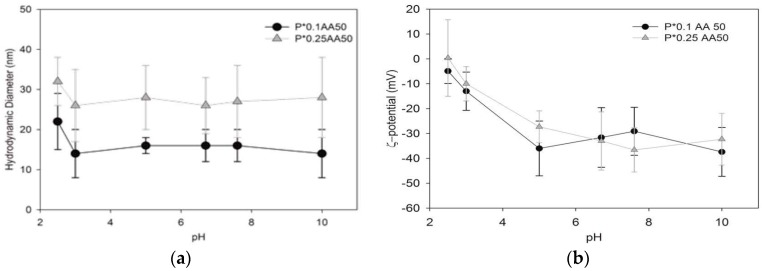
(**a**) Hydrodynamic diameter and relative standard deviation for P*0.1AA50 and P*0.25AA50 reported at the variance of pH and constant ionic strength (1 mM); and (**b**) ζ-potential and relative standard deviation for P*0.1AA50 and P*0.25AA50 reported at the variance of pH and constant ionic strength (1 mM).

Colloidal stability of aqueous dispersions of the bare NGs was evaluated by DLS measurements during storage at 4 °C for 24 months. NGs hydrodynamic size and surface charge density have been measured at different pH (2.5–10) and controlled ionic strength (1 mM). DLS data for two representative systems are shown in Figure 2. Hydrodynamic diameters and relative PDI are fairly invariant between pH 4 and 10. At pH < 3.5 nanoparticles become slightly bigger due to the onset of aggregation phenomena. Below pH 2.5, aggregation leads to precipitation, thus impairing the measurement. This is because at pH < p*K*_a_ for AA (p*K*_a_ = 4.75) a great proportion of NGs carboxyl groups are protonated and ζ-potential values become around 0 mV (see Figure 2). Therefore, the contribution to stabilization from electrostatic repulsion among carboxyl anions ceases. At pH > 2.5, the ζ-potential values become negative and their absolute values increase with pH due to progressive dissociation of carboxyl groups. Since the degree of dissociation of carboxyl groups does not affect NGs hydrodynamic size, but it does affect their surface charge density, we expect these groups to be preferentially located at the terminus of NGs dangling arms and/or attached to the crosslinked core.

The degree of conjugation of the fluorescent probe (AF) and Folic Acid (FolAc) are reported in Table 3. No significant variation of hydrodynamic size is observed. AF causes a slight reduction of surface charge density, while Folic Acid does not.

A sequential conjugation was performed to generate NGs labeled with AF and decorated with Folic Acid. The selected NG system was P*0.5AA50, since it showed the highest conjugation degrees for both AF and Folic acid. For the double conjugated systems, the reaction with AF was performed first and in under-stoichiometric conditions. The degree of conjugation with folic acid is reduced to about 50. NGs dimensions after each conjugation step were checked via DLS in order to confirm that the hydrodynamic size is not affected by the conjugations sequence. Furthermore, the dispersibility in buffer of multifunctional NGs was also evaluated. In particular, the freeze-dried NGs showed hydrodynamic sizes very similar to their parent systems (as produced) upon re-hydration.

### 2.2. Folate-Targeting Studies In Vitro

**Figure 3:**
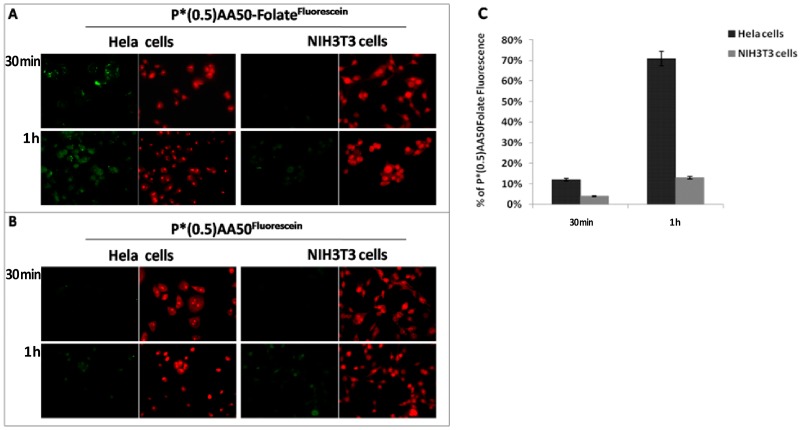
NGs targeting studies: P*(0.5)AA50-Folate^fluorescein^ (**A**); and P*(0.5)AA50^fluorescein^ (**B**) localization studies by fluorescence microscopy; and P*(0.5)AA50-Folate^fluorescein^ quantitative uptake studies by flow cytometric analysis (**C**) on Hela and NIH3T3 cells (30 s and 1 h). (Fluorescence images: NGs = green, Cells = red).

Many tumor cell lines, like Hela cells, overexpress folate receptor (FR), consequently folate conjugated to nanoparticles can confer them specific tumor targeting property. For this purpose, NGs conjugated with Amminofluorescein (AF) (P*(0.5)AA50^fluorescein^) and with FolAc (P*(0.5)AA50-Folate^fluorescein^) were used. Nanoparticles targeting ability was investigated by placing both Hela (a positive FR cell line) and NIH3T3 cells (a negative FR cell line) in the same culture environment (as reported in M&M). Therefore, the specific tumor cellular internalization of the Folate-derivated nanogels was investigated in both cell lines after 30 min and 1 h. Nanosystems without folate were used as control. As shown in Figure 3, P*(0.5)AA50-Folate^fluorescein^ uptake appeared completely different for the two cell lines analyzed; in fact, just in 30 min of incubation, Hela cells showed considerably higher nanoparticles uptake than NIH3T3 cells, in which the green signal relative to NGs was completely absent (Figure 3A). Moreover, it was evident how folate-conjugate nanogels specifically were localized as green spots on the plasma membrane of Hela cells, suggesting a specific interaction with FR. Conversely, NIH3T3 cells showed widespread fluorescence after 1 h of incubation.

On the other hand, in both cell lines incubated with P*(0.5)AA50^fluorescein^ was evident that the green fluorescence, relative to P*(0.5)AA50^fluorescein^ uptake, appeared only after 1 h of treatment and it resulted lower and spreader in the cytoplasm. In order to quantify the relative internalization rates of the folate-conjugate, flow cytometric analysis were carried out as shown in Figure 3C. Histograms relative to Hela cells showed a significant higher fluorescence intensity respect to those of NIH3T3 cells, after 1 h of incubation (70% vs. 15%, respectively). Thus, according to previous co-culture experiment, it was evident that the targeting strategy strongly enhanced the selective cellular nanosystems uptake.

### 2.3. Physicochemical Characterization of the P*(0.5)AA50-AEDP-DOX Conjugate

**Figure 4:**
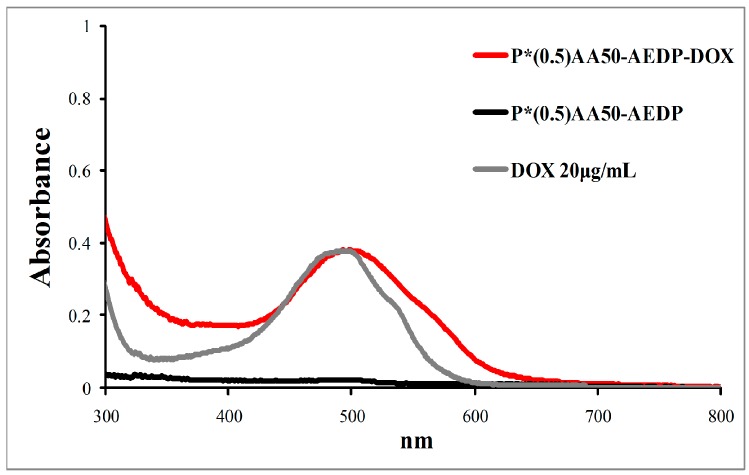
UV-vis absorption spectra of P*(0.5)AA50-AEDP-DOX, P*(0.5)AA50-AEDP and free doxorubicin (20 μg/mL).

To create a GSH-responsive nanodrug, a redox-sensitive spacer, named 3-(2-aminoethyl) dithiopropionicacid (AEDP), was inserted between nanogels and the anticancer drug doxorubicin (DOX). Indeed, AEDP is a small molecule with a disulfide bridge, that makes it sensible to the redox state of the microenvironment, and primary amino group and carboxylic extremities that allow the coupling of the drug to the nanogels. The conjugation was carried out by a standard protocol to obtain P*(0.5)AA50-AEDP-DOX conjugates (see Materials and Methods). The amount of conjugated DOXO was estimated through spectrophotometric analysis. In Figure 4, the UV-Vis absorption spectra of P*(0.5)AA50-AEDP-DOX, P*(0.5)AA50-AEDP and free drug are shown. It can be noticed that the conjugated nanosystem shows an absorbance peak at 490 nm that it is similar to that of the free drug, although somewhat shifted, while it is absent in non-conjugated system. The quantification of the amount of drug incorporated in the NGs was then performed using a calibration curve of the free drug. In particular, the measured concentration of DOX in the P*(0.5)AA50-AEDP-DOX system is 24 μg/mL. The dispersion of P*(0.5)AA50-AEDP-DOX nanogels behaves similarly to the parent bare nanogel, such as its size and colloidal stability.

### 2.4. Drug Release Study

**Figure 5:**
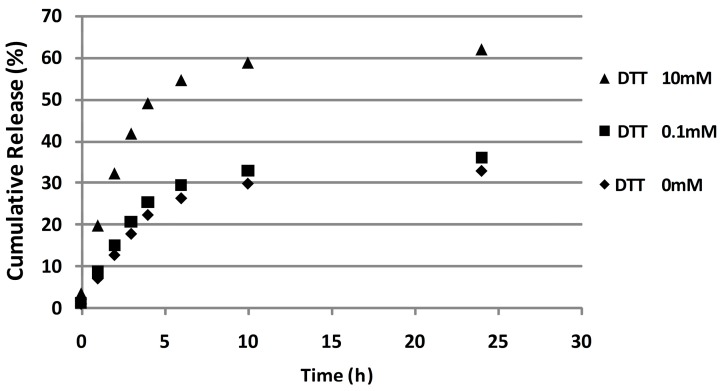
In vitro release of DOX from P*(0.5)AA50-AEDP-DOX in 1 mM Tris-HCl buffer (pH 7.4 at 37 °C ), with or without DTT (10 mM, 0.1 mM, and 0 mM) treatment.

Nanodrug responsiveness was investigated by in vitro release experiments that were carried out in media simulating different body compartments, by treating P*(0.5)AA50-AEDP-DOX systems with DTT, as reducing agent. Doxorubicin release in external medium was monitored by absorbance readings at 490 nm at selected time until 24 h (Figure 5). No burst effect is evident.

As expected, a remarkable redox-dependent release profile can be noticed at DTT 10 mM. In particular, after 12 h about 60% of doxorubicin payload was slowly released in the high reductive environment, while only 30% of doxorubicin was released at low reducing agent concentration.

DOX release profile was also monitored in the absence of DTT as control. A trend similar to that found for the low reductive conditions was observed. These results demonstrate that the amount of drug released in 24 h at DTT 10 mM is about two-fold higher than the control.

### 2.5. Intracellular Drug Release Studies of P*(0.5)AA50-AEDP-DOX

**Figure 6:**
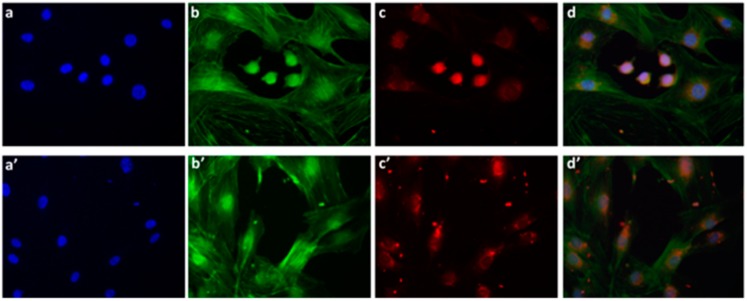
MC3T3-E1 cells pretreated (**a**–**d**) or not (**a′**–**d′**) with GSH-OET and then incubated with P*(0.5)AA50-AEDP-DOX for 3 h (Nuclei = Dapi blu (**a**,**a′**), Cytoskeleton = FITC-phalloidin green (**b**,**b′**), DOX = Doxorubicine red (**c**,**c′**)). Magnification 40×.

P*(0.5)AA50-AEDP-DOX cellular uptake and intracellular drug release were investigate using, as model systems, MC3T3-E1 cells and Hela cells, which respectively mimicked the normal and cancerous state. In order to mime the stress condition, both cell lines were pretreated for 2 h with 10 mM of glutathione monoester (GSH-OET) that can penetrate plasma membrane and rapidly induce an increase of GSH intracellular concentration through ethyl ester hydrolyzation. Therefore, cells were treated with P*(0.5)AA50-AEDP-DOX for 3 h. GSH-OET-untreated cells, but incubated with the same amount of NGs, were used as control. GSH-OET effect and therefore GSH activity were evident in epifluorescence microscopy images relative to normal MC3T3-E1 cells stained with DAPI for nuclei and phalloidin-FITC for actin cytoskeleton. In Figure 6, a different intracellular trafficking and red fluorescence intensity of DOX in cells pretreated or not with GSH-OET was evident. Indeed, in GSH-OET pre-treated cells the doxorubicin red fluorescence appeared localized exclusively in the nuclei (Figure 6a–d), while it appeared muffled and punctuate in cytoplasm of untreated cells and partially in the nuclei (Figure 6a’–d’). Indeed, doxorubicin effect was also evident by the presence of some cells with a spherical morphology (Figure 6a–d), typical of apoptotic cells, devoid of cell-cell contact.

**Figure 7:**
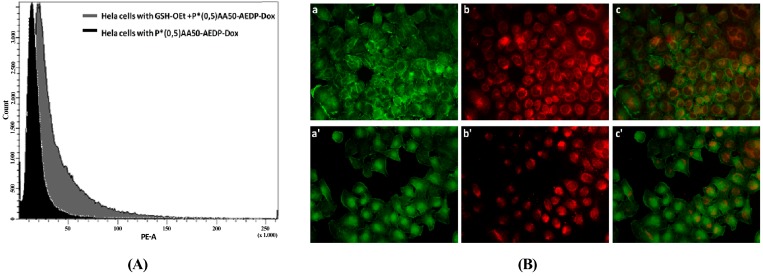
(**A**) Flow cytometric plot of fluorescence intensity of Hela cells pretreated or not with GSH-OET and incubated with P*(0.50)AA50-AEDP-DOX for 3 h; and (**B**) CLSM observation of Hela cells pretreated with GSH-OET (**a**–**c**) and without GSH-OET (**a’**–**c’**) and incubated with P*(0.5)AA50-AEDP-DOX for 3 h (Cytoskeleton = green phalloidin FITC, DOX = red). Magnification 40×.

Figure 7B shows the same uptake experiment relative to Hela cells, by confocal microscopy. Cytoskeleton of GSH-OET pretreated or not Hela cells was stained with phalloidin^FITC^. In both cases, P*(0.50)AA50-AEDP-Dox were internalized and DOX was released to reach cell nuclei. Importantly, DOX fluorescence was brighter when Hela was pretreated with GSH-OET, and it was possible to note apoptotic cells. On the other hand, in contrast with normal cells, doxorubicin also appeared in the nuclei of GSH-OET untreated cells, probably because of the basal cytosolic reducing condition of such cancerous cells like Hela cells. On the other hand, data relative to flow cytometric analysis of Hela cells (Figure 7A) showed an evident histogram shift to the direction of high fluorescence intensity of the GSH-OET pretreated cells respect to untreated ones. Therefore, these results could suggest an enhanced DOX release in tumor cells due to a major degradation of pendant disulfide linkages in response to high GSH concentration. 

### 2.6. In Vitro Cytotoxicity of P*(0.50)AA50-AEDP-DOX

**Figure 8:**
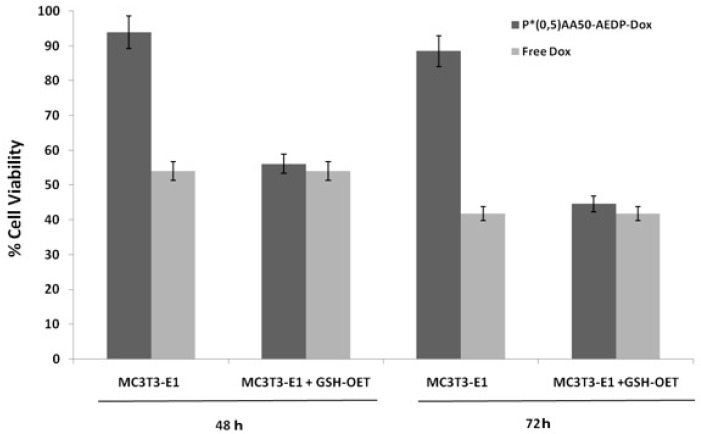
Cytotoxicity assay on MC3T3-E1 cells pretreated or not with GSH-OET and incubated for 48 and 72 h with P*(0.5)AA50-AEDP-DOX or free DOX. Cell viability expressed as percentage of untreated cells.

P*(0.50)AA50-AEDP-DOX cytotoxicity was investigated by Alamar blue assay both in MC3T3-E1 and Hela cells, pretreated with 10 mM GSH-OET for 2 h. Noteworthy, the pretreatment with GSH-OET did not affect the proliferation of MC3T3-E1 and Hela cells in our experiment (data not shown). Moreover, both cell lines without the GSH-OET pretreatment or incubated with free DOX were used as controls. Non-pretreated MC3T3-E1 cells did not exhibit significant cell proliferation inhibition when incubated with P*(0.50)AA50-AEDP-DOX for 48 h. Furthermore, cell viability still remained higher than 90% even after 72 h of treatment (Figure 8).

**Figure 9:**
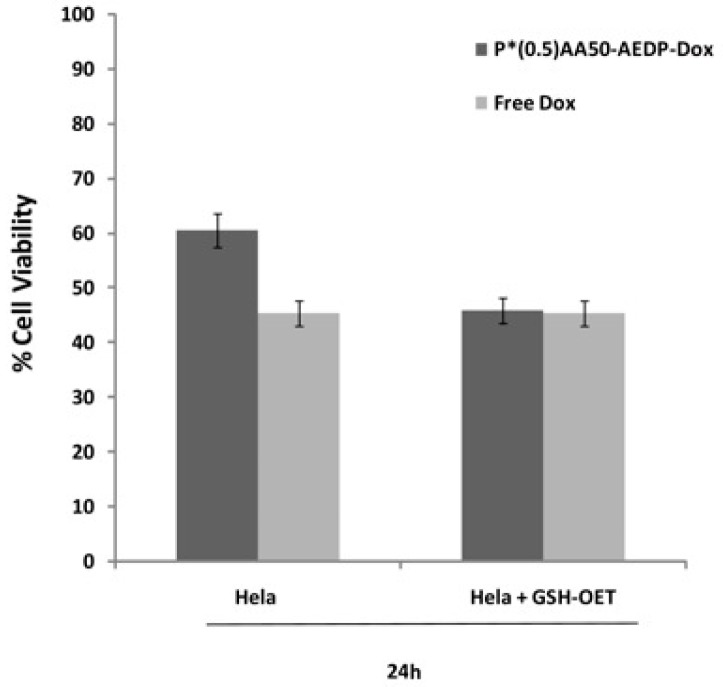
Cytotoxicity assay on Hela cells pretreated or not with GSH-OET and incubated for 24 h with P*(0.5)AA50-AEDP-DOX or free DOX. Cell viability expressed as percentage of untreated cells.

Conversely, GSH-OET pretreated cell viability decreased significantly (50% of cellular growth inhibition) after 48 h, and further it decreased within 72 h. In addition, the same viability values were obtained for MC3T3-E1 cells treated with the same concentration of free drug. These results demonstrated that high intracellular GSH levels induced P*(0.5)AA50-AEDP-DOX destabilization and consequently DOX release; in this way, cell proliferation inhibition was enhanced, carrying out a similar cytotoxicity of free drug. By contrast, in physiological redox condition, nanosystems did not affect cell viability, even after 72 h of incubation. The redox-induced drug release was more evident in tumor cells, such as Hela cells; indeed, in Figure 9, it was evident that Hela cell viability decreased with both nanodrugs and free DOX, just after 24 h.

In order to obtain the maximum drug release, cells were pre-treated with GSH-OET and incubated with P*(0.5)AA50-AEDP-DOX. Data showed that cell viability was lower respect non-pretreated ones, but at the same level of the control (free drug). 

### 2.7. Conjugation of Bcl-2 siRNA to P*(0.5)AA50-AEDP

**Figure 10:**
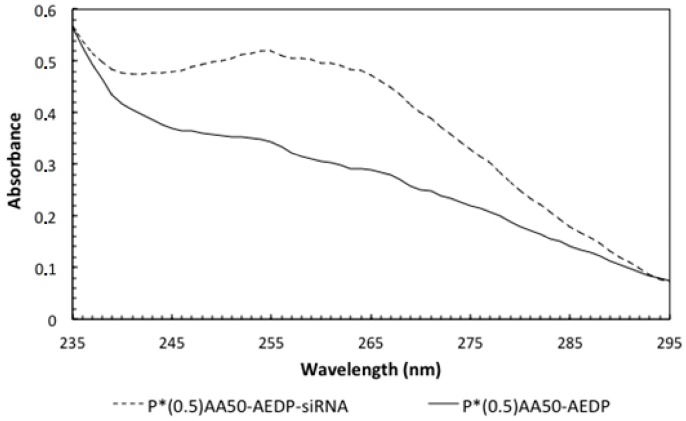
UV-vis absorption spectra of P*(0.5)AA50-AEDP alone or conjugated with Bcl-2 siRNA.

P*(0.5)AA50-AEDP nanogels were also conjugated with small oligonucleotides, like Bcl-2 siRNA, carrying an amino group at one end (5′-AminoC6linker modified Bcl-2 siRNA) by EDC and Sulfo-NHS standard protocol. Absorption spectra reported in Figure 10 show that the siRNA-conjugated NG spectrum has the characteristic nucleic acid peak at 260 nm, while no absorption peak was observed in the nonconjugated system, used as control.

The amount of siRNA conjugated to the nanoparticles was determined by a spectrophometric analysis, using a standard curve of free siRNA (Data not shown). By interpolating their optical density to the standard trend, it was possible to calculate that 5.6 ng/µL of 5′-AminoC6linker modified Bcl-2 siRNA were conjugated to P*(0.5)AA50-AEDP.

### 2.8. P*(0.5)AA50-AEDP-siRNA Effect on Bcl-2 Protein Expression

**Figure 11:**
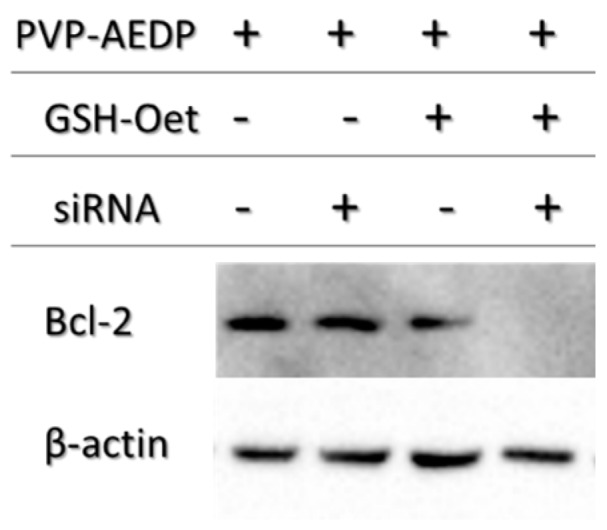
Western blot analysis of Bcl-2 expression in Hela cells.

Finally, Western blot analysis determined the GSH-mediated effect of P*(0.5)AA50-AEDP-siRNA on Bcl-2 protein expression. To test a specific release mechanism, Hela cells were pre-treated with GSH-OET and incubated with P*(0.5)AA50-AEDP-siRNA nanogels for 48 h. GSH-OET untreated cells and incubated with P*(0.5)AA50-AEDP alone or conjugated with the oligonucleotide were used as control. Western blot analysis (Figure 11) shows that without GSH-OET treatment, there was not a significant silencing of the target protein; indeed, the intensities of Bcl-2 bands were very similar. By contrast, in samples subjected to the action of the GSH inductor, substantial difference was evident. Indeed, in the specimen incubated with PVP-AEDP-siRNA NGs, the band relative to Bcl-2 protein was almost totally disappeared respect to the negative control (cells incubated with PVP-AEDP NGs). β-actin was used as a protein loading control for comparison of the Bcl-2 protein expression over time.

## 3. Discussion

In the recent years, active tumor-targeting drug delivery systems have attracted particular interest in cancer treatment, since the conventional chemotherapy lacks specificity against cancer cells and is associated with significant side effects. Functionalized nanoparticles have been shown to have a potential to recognize cancer and accumulate drugs in tumor cells, by conjugating specific molecules to realize this objective [18]. In previous works, we described a physico-chemical and biological characterization of PVP nanogels like size, shape, biocompatibility, oxidative stress induction, the ability to cross biological membranes, genotoxicity and the easy surface-decoration, qualifying them as potential therapeutic nanocarriers [13,14,15,16,17]. In this study, we initially investigated if the conjugation of NGs with folic acid could improve its tumor targeting ability. Cancer cells are highly dependent on folate for DNA synthesis and cell division, and they express high levels of FR, compared to normal cells. We exploited this differential expression of folic acid receptors on cancer and normal cells in a co-culture system of Hela and NIH3T3 cells respectively, demonstrating that the conjugation of nanogels with folic acid increase the cellular uptake in cancer cells (Figure 3). Even flow cytometric analysis suggested a fundamental folate role for tumor specific targeting; indeed, it increased NGs affinity for Hela cells through a specific ligand-receptor recognition. One of the mechanisms for the higher efficacy might be upper intracellular accumulation of chemotherapeutics linked to nanoparticles, exploiting a controlled release. Therefore, once established the nanosystems targeting ability, we focused our attention to perform a drug controlled release mechanism, basing on specific tumor cell characteristics like redox state determinate by glutathione (GSH) concentration [10]. The proposed action mechanism provides the creation of a “stimulus-responsive” nanodrug, sensitive to redox environment changes: at normal reducing condition, such as the extracellular microenvironment or in the body stream, the drug remains hooked to the nanogels. Conversely, when nanogels cross the cellular membrane, in presence of high glutathione levels, as observed in cancer cells, the breaking of the sulfide bridge occurs and drug is release into the cytosol [12]. In this view, we selected chemotherapeutic drug doxorubicin as model because of its significant anticancer activity, its chemical structure that allows both the bind with nanogels and follow its trend by red autofluorescence. To develop a glutathione-mediated delivery, drug was linked to nanogels through AEDP spacer containing a cleavable disulfide bridge. P*(0.5)AA50-AEDP-DOX system was prepared and characterized by size and drug loading. 

To investigate whether the disulfide bridge is degradable in reducing environments, DOX release profiles were determined in the presence of different concentrations of reducing agent. In particular, 10 mM and 0.1 mM of DTT solution were used to mime the cytoplasm reductive environment of cancer cells and physiological extracellular fluid, respectively. P*(0.5)AA50-AEDP-DOX was able to release twice as much doxorubicin under the reductive stimulus which matches the intracellular cancer GSH concentration (10 mM), by inducing the sulfhydryl bond reduction of the spacer. As expected, in vitro DOX release was highest in reducing condition, which matches with the intracellular cancer GSH concentration (10 mM).

We also examined the P*(0.5)AA50-AEDP-DOX intracellular release in normal and cancer cells (MC3T3-E1 and Hela cells) and the enhanced intracellular DOX release was observed in glutathione monoester (GSH-OEt) pretreated cells. These data suggested that the cytoplasmic glutathione increase could induce disulfide bridge degradation of AEDP linker and consequently the intracellular DOX release from P*(0.5)AA50-AEDP-DOX, as indicated by doxorubicin presence in nuclei and therefore its intercalation into DNA (Figure 6). By contrast, MC3T3-E1 cells without GSH-OET pre-treatment emitted weak and spotted red DOX fluorescence in the cytoplasm surrounding the nuclei and no evidences of apoptotic events related morphology were observed; thus, it could be hypothesized that DOX remained linked to nanogels, due to the low physiological reductive environment. Moreover, even if glutathione concentration in tumor cells is higher respect to normal ones, several reports also describe GSH-OET pretreatment of cancer cells to enhance cellular GSH levels [21]. Effectively, confocal microscopy and flow cytometric analysis shown a different drug release profile by comparing pretreated or not tumor cells. Furthermore, the redox responsive drug release also induces higher DOX efficiency as shown by higher cytotoxicity in GSH-OET pretreated Hela and MC3T3E1 cell lines. In relation to cytotoxicity data, it is possible to suppose that the disulfide bridges are stable at physiological condition, but respond to high reductive conditions (GSH) via reversible cleavage into free thiols, triggering a fast DOX release in cytoplasm. Therefore, the glutathione-mediated drug delivery of P*(0.5)AA50-AEDP-DOX is able to realize the rapid intracellular drug release and enhance the growth inhibition to tumor cells. The possibility to easily functionalize these kinds of nanoparticles with molecules of different nature (like folate, aminofluorescein, doxorubicin, and small oligonucleotides) make them optimal candidate for siRNA delivery. Small oligonucleotides, like siRNA, are very unstable and can be easily degraded in bloodstream or in the extracellular matrix. Therefore, their conjugation with PVP nanogels could protect them and target to the specific site [14]. For this purpose, we choose a siRNA for Bcl-2, the first anti-death gene discovered, implicated in tumor progression. RNAi preliminary studies had shown P*(0.5)AA50-AEDP-siRNA propensity as siRNA delivery system; indeed, they are able to link modified siRNA without any alteration of their silencing functionality after has been released, as indicated by Western blot analysis. All these data suggest the possibility to develop an innovative therapeutic approach for tumor therapy. Evidently, the progress in the development of these smart nanogels and their prodrugs version will keep by in vivo studies in order to better validate their properties. The introduction of stimuli-responsive drug delivery system should be considered a good way to improve nanomedicine progress. Moreover, the opportunity to obtain a specific tumor targeting and to trigger drug release systems can be considered an innovative nanomedicine device. The future of our current work will be directed to identify new therapeutic targets to extend the use of these nano-systems in various human diseases observing their behavior in vivo, using mouse model. The simultaneous conjugation with different bio-molecules could improve their efficiency not only concerning tumor therapy, but also neurodegenerative pathologies.

## 4. Materials and Methods

### 4.1. Materials

Aminofluorescein (AF), Folic Acid (FolAc), 1-ethyl-3-(3-dimethylaminopropyl)carbodiimide (EDC), *N*-Hydroxysulfosuccinimide (Sulfo-NHs), 2-(*N*-morpholino)ethanesulfonic acid (MES), glutathione (GSH), Nickel(II) chloride hexahydrate, 4-(2-Hydroxyethyl)piperazine-1-ethanesulfonic acid (HEPES), Pyrocatechol Violet (PV) were supplied by Aldrich and were used without further purification. 3-(2-aminoethyl) dithiopropionicacid (AEDP) was supplied by VWR. Poly(*N*-vinyl pyrrolidone)-co-acrylic acid nanogels (NG) were synthetized by electron beam irradiation and used as substrates [20]. Systems are named as P*(X)AAY, where X is the weight percent of polymer in solution and Y is the molar ratio between the PVP repeating unit in the polymer and the acrylic acid in the irradiated solution (nominal concentration of carboxyl groups in the system). The actual concentration of carboxyl groups either formed by radiation-induced oxidation of PVP or grafted with AA was determined by colorimetric method that is based on Nickel complexation [19]. The primary amino groups present in the nanogels were estimated were titrated upon reaction with fluorescamine [19].

### 4.2. Preparation of NG-Fluorescein, NG-Folic Acid, NG-Doxorubicin and NG-siRNA Variants

NGs were labeled with a fluorescent probe, AF, using an EDC/Sulfo-NHS protocol in order to activate the carboxyl groups of the nanogels [20]. A given volume of NG aqueous dispersion was mixed with EDC and Sulfo-NHS aqueous solution (MES pH 5.5) for 30 min, then a given amount of AF was added, keeping constant the molar ratio between the nominal concentration of carboxyl groups in the system and the probe added to the reactor feed (0.25 and 10). The reaction was conducted for four hours under gentle stirring at room temperature in dark conditions. The determination of the amount of probe conjugated to the nanogel was carried out by spectrophotometer analysis. While bare NGs do not show any absorption band in the wavelength range where AF absorbs, the AF-conjugated systems show the absorption band of AF, with a red-shift (+5 nm). Since AF can also be physically entrapped into the hydrophobic pockets created by crosslinking, nanogels incubated with AF in the same conditions as for the conjugation, but without EDC/sulfo-NHS, were prepared as a control and subjected to the same release experiment. While all entrapped AF was released after 48 h by the control, a percentage of AF was retained by the conjugated systems. The amount of conjugated AF was calculated as the difference between the amount of probe in the uptake solution and the amount of probe released after 48 h. Samples for biological analysis were dialyzed for longer than 48 h in dark conditions.

**Figure 12:**
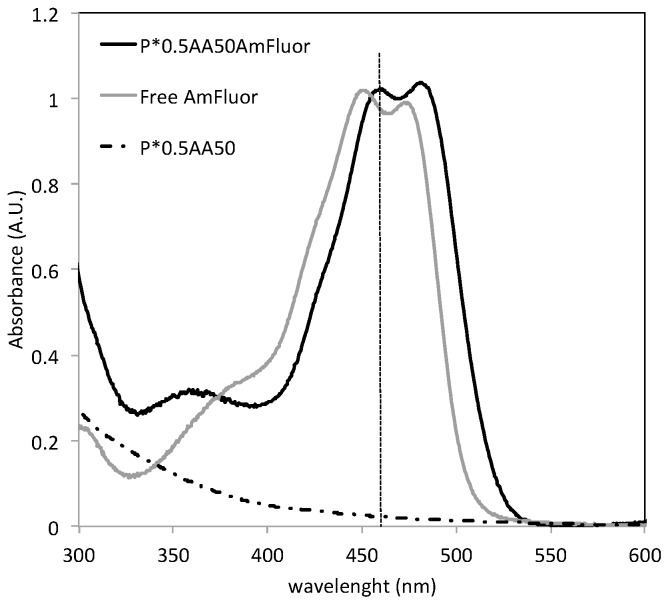
Normalized absorption spectra for a typical conjugated NG system, the free AmFluor solution and the bare P*0.5AA50 NGs.

Conjugation with folic acid was performed using the same EDC/Sulfo-NHS protocol used for AF In consideration of the fact that the NGs have primary amino groups, folic acid is likely to be coupled to the amino groups of the nanogels via reaction of one the carboxyl groups [22]. The product was purified by dialysis against water, using 100 kDa cutoff dialysis tubes. The conjugation degree was determined by spectrophotometric measurements of the dialyzed product (Figure 12).

For the synthesis of doxorubicin-conjugated variants, P*0.5AA50 NGs were selected and conjugated first with AEDP and then with doxorubicin hydrochloride (DOX, Ebewe pharma). For both conjugation reactions, the standard EDC/Sulfo-NHS protocol was used. Reaction and purification conditions after each modification step were the same as those used for AF conjugation. 

For the first step, the amount of AEDP was scaled down in order to minimize interparticle bridging. In these conditions, AEDP homopolymerization was tested to be negligible.

For siRNA-conjugation, P*(0.5)AA50 nanogels were dialyzed (14 kDa) against NaOH 0.2 M for 24 h in order to deactivate Ribonuclease (RNAse) that could degrade the siRNA, and then dialyzed against H_2_O RNAse free. The standard EDC/Sulfo-NHS conjugation protocol was adopted. The blend was incubated for 30 min in agitation at room temperature. Therefore, 720 pmol of 5′-AminoC6linker Bcl-2 siRNA (Qiagen, Hilden, Germany) were mixed and incubated for 3 h in agitation at 4 °C and then dialyzed (14 kDa) against H_2_O RNAse free to eliminate the excess of unlinked siRNA.

Conjugation degrees of the various ligands were estimated by UV-visible absorption measurements at room temperature with Shimadzu 2401-PC spectrofluorimeter (Shimadzu Corporation, Kyoto, Japan) (scan speed 100 nm/min, integration time 2 s, bandwidth 1 nm) and for NGs conjugated with doxorubicin also with a DU-730 Life Science spectrophotometer, Beckman Coulter (Brea, CA, USA). For RNAi experiments, the samples were then dialyzed against the appropriate cell colure medium. The amount of siRNA conjugated to NGs was determined by reading the absorbance values at 260 nm employing the NanoDrop 1000 Spectrophotometer (Thermo Scientific, Waltham, MA, USA). Different concentrations of free siRNA were used for a standard assay.

### 4.3. Physico-Chemical Characterizations

The hydrodynamic dimensions of the differently conjugated NG variants were measured by dynamic light scattering (DLS) using a Brookhaven Instruments BI200-SM goniometer (Brookhaven Instruments Corporation, Holtsville, NY, USA) equipped with a 50 mW He-Ne laser tuned at λ = 632.8 nm. Measurements were carried out placing samples in a quartz cell within the thermostated cell compartment of the instrument at 25 ± 0.1 °C and at 90° scattering angle after filtration (pore size 0.45 μm). In consideration of their monomodal size distribution, DLS data were analyzed according to the method of cumulants [17,23,24,25]. Ζ-potential measurements were acquired at 25 °C using a Malvern Zetasizer Nano-ZS (Malvern instrument Ltd., Malvern, UK) equipped with a He–Ne laser at a power of 4.0 mW. Systems are characterized by a monomodal ζ-potential distribution; the mean ζ-potential and the relative distribution width are reported in Table 1. For zeta-potential measurements systems were dialyzed against water. Atomic force microscopy (AFM) was performed on a selected aqueous system (5 ng/mL) deposited on mica substrates modified with 3-aminopropyl triethoxysilane (APTES) [15]. Samples have been examined under water in tapping mode at 2 Hz scanning rate using a MMFP3D AFM (Park Systems Corporate Headquarters, Suwon, Korea) and MSNL D tips (Bruker Nano Inc., Billerica, MA, USA) with nominal resonance frequency of 10–20 kHz and spring constant k ~30 pN/nm.

### 4.4. Co-Culture Experiment

Human cervical cancer (Hela) and mouse embryo fibroblast (NIH3T3) cells were grown in Dulbecco’s Modified Eagle Medium (DMEM) containing 10% (*v*/*v*) fetal bovine serum (FBS, EuroClone, Milan, Italy), 100 units per mL penicillin G, 100 µg·mL^−1^ streptomycin (EuroClone) and 2 mM l-glutamine (EuroClone) at a density of 5 × 10^3^ cells/well into 12 well/plates containing sterile glass coverlips, for 24 h at 37 °C in a humidified atmosphere of 5% CO_2_. Following, the two slides, everyone with each cell line seeded on, were placed in the same culture environment and incubated with P*(0.5)AA50-Folate^fluorescein^ nanogels (45 μg/mL) for 30 min or 1 h. Equivalent conditions were used as control: both cell lines were treated with the same amount of nanocomplex without folate (P*(0.5)AA50^fluorescein^). After the established incubation times, cells were washed twice with complete phosphate buffer saline (PBS, EuroClone), fixed with 3.7% formaldehyde for 15 min and again washed twice with PBS. Afterwards, cells were stained with ethidium bromide (1:1000 in PBS) for 1 min at room temperature. Fluorescence within the cells was evaluated by means of epifluorescence microscopy (Leica DFC3000 G, Wetzlar, Germany).

### 4.5. Internalization Studies by Flow Cytometry

Hela and NIH3T3 cells were cultured in a 6-well plates in complete DMEM medium until 70% of confluence. After, both cell lines were incubated with P*(0.5)AA50-Folate^fluorescein^ nanogels (45 μg/mL), for 30 min and 1 h. After washing with PBS without Ca^2+^ and Mg^2+^, cells were detached by Trypsin-EDTA 1× in PBS (EuroClone) and collected by centrifugation at 1000 rpm for 5 min. Therefore, cellpellets were resuspended in 0.5 mL of PBS buffer and analyzed by flow cytometric analysis, using a FACS-Calibur (Becton Dickinson, Heidelberg, Germany). For each sample, data of 1 × 10^4^ gated events were collected and analysis was performed by BD FACS diva software.

### 4.6. In Vitro Drug Release Measurement

P*(0.5)AA50-AEDP-DOXnanogels (5 mL) were placed into dialysis membranes (MWCO 12 kDa) and each tube was dipped in 50 mL of buffer (1 mM Tris-HCl pH 7.4) alone ( as control) or containing 10 or 0.1 mM of DTT (Dithiothreitol, Sigma Aldrich, Saint Louis, MO, USA).Every system was maintained at 37 °C under continuous shaking condition (200 rpm). At predetermined time (0, 1, 2, 4, 6, 10, and 24 h), 10 mL of external buffer solution was withdrawn and replaced with 10 mL of fresh buffer. Doxorubicin percentage released was determined by using spectrophotometer measurements (490 nm) (Glomax^®^-Multi Detection System, Promega, Madison, AL, USA).

### 4.7. Intracellular DOX Release Studies

Intracellular drug release experiments were performed by flow cytometry and analyzed by confocal and epifluorescence microscopy.

#### 4.7.1. FACS analysis

Hela cells were seeded in 6-well plates, until the confluence state. Subsequently, cells were treated with glutathione reduced ethyl ester (GSH-OET, Sigma Aldrich) (10 mM) for 2 h [26]. After washing with PBS, cells were incubated at 37 °C for 3 h with P*(0.5)AA50-AEDP-DOXnanogels at final DOX concentration of 1 μg/mL in complete DMEM medium. GSH-OET untreated cells were used as control. Next, cells were washed twice with PBS, detached by Trypsin-EDTA 1×, collected by centrifugation at 1000 rpm for 5 min order to obtain cell pellet that was re-suspended into 0.5 mL of PBS. For each sample were collected 1 × 10^4^ events investigated by FACS-Calibur (Becton Dickinson) using BD FACS Diva software.

#### 4.7.2. Fluorescence observation

Mouse Osteoblastic cell line (MC3T3-E1) and Hela cells were seeded, at a density of 12 × 10^4^ cells per well, in a 12-multiwell slides containing sterile covers lip in complete DMEM. After 24 h of growth at 37 °C, a sample of each type of cells was treated with 10 mM GSH-OET for 2 h. Cells were washed by PBS and incubated for 3 h at 37 °C with the P*(0.5)AA50-A EDP-DOX nanogels, at final DOX concentration of 1 μg/mL in complete DMEM. Cells without GSH-OET treatment were used as a control. Following, both cell lines on the slides were fixed with 3.6% formaldehyde for 15 min and rinsed with PBS for three times. The actin cytoskeleton was labeled with phalloidin^FITC^ (1:500 in PBS) for 30 min at room temperature. After 3 washes with PBS, MC3T3-E1 cells were stained with DAPI (1:1000 in PBS) for 30 s, at room temperature for marking the nuclei in blue. Finally, the MC3T3-E1 slides were mounted and observed by epifluorescence microscope (Leica, Wetzlar, Germany), while the Hela slided with confocal microscopy (CLSM, Olympus IX70 laser system with MellesGriot, Shinjuku, Tokyo, Japan).

### 4.8. Cytotoxicity Assay

P*(0.5)AA50-AEDP-DOX cytotoxic effect was evaluated on MC3T3-E1 and Hela cells by Alamar Blue assay (Sigma Aldrich). Both cell lines were seeded into a 96-multiwell at 6 × 10^3^ cells per well, in complete DMEM for 24 h and then treated with 10 mM GSH-OET for 2 h at 37 °C. Therefore, Hela cells were incubated with P*(0.5)AA50-AEDP-DOX nanogels or free DOX as control at the final DOX concentration of 1 μg/mL for 24 and 48 h; while MC3T3-E1 cells were treated with drug alone (as control) or conjugated with NGs at the final DOX concentration of 0.3 μg/mL for 48 and 72 h. Cells GSH-OET untreated and incubated with NGs were used as another control. After treatment, cells were incubated with Alamar Blue solution (10% in culture medium), a reagent for evaluating cellular health, for 4 h at 37 °C. Fluorescence intensity (λexc 530/25 nm and λemm 590/35 nm) was read through spectrofluorometer (SinergyHT, BioTek, Winooski, VT, USA), which changes according to the degree of cell viability. The results were expressed as the percentage ratio of the fluorescence between the treated sample with respect to untreated cells, used as negative control.

### 4.9. Treatment with NG-AEDP-siRNA Complex

Hela cells were seeded in 6-well plates and grown at 37 °C with 5% CO_2_ until the confluence state. Some samples were incubated for 2 h at 37 °C with 10 mM of GSH-OET in complete medium. After the opportune washes with PBS, P*(0.5)AA50-AEDP or P*(0.5)AA50-AEDP-siRNA (300 pmol of siRNA) complex were added for 48 h.

### 4.10. Extraction of Soluble Proteins and Western Blot Analysis

Hela cells were seeded in 6-well plates maintained at 37 °C in a humidified atmosphere of 5% CO_2_. After the appropriate treatment, the samples were washed with PBS without Ca^2+^ and Mg^2+^, detached by Trypsin-EDTA 1× in PBS and collected by centrifugation at 1000 rpm for 5 min. The pellets were re-suspended in an appropriate volume (depending of the pellet size) of RIPA Buffer 1× (50 mM Tris-HCl pH 7.5; 0.5% sodium deoxycholate; 150 mM NaCl; 1% Triton X-100) supplemented of proteases inhibitors (1 mM of phenylmethylsulfonyl fluoride (PMSF); 1 µM of Pepstatin A; 100 µM Leupeptin; 10 mM Ethylenediaminetetraacetic acid (EDTA)). Therefore, samples were incubated in ice for 15 min and constantly subject to micro vortex mixer treatments and then centrifuged at 10,000 rpm for 20 min. The amount of proteins extracted presented into the supernatant were calculated by the Bradford assay (Bradford Reagent, Sigma Aldrich), using different concentrations of bovine serum albumin (BSA) as Standard Assay. Twenty micrograms of proteins were mixed with sample buffer 1× (62.5 mM Tris-HCl pH 6.8; 2.5% SDS; 0.002% Bromophenol Blue; 0.7135 M (5%) β-mercaptoethanol; 10% glycerol) and incubated at 100 °C for 5 min and immediately situated in ice. The proteins were separate by 15% sodium dodecyl sulfate Polyacrylamide gel electrophoresis (SDS-PAGE). The proteins were transferred from the gel to a nitrocellulose membrane (Hybond, Amersham) through electroblotting at 100 V and 300 mA for 90 min at low temperature, in transfer buffer (20% methanol; 10% Running buffer 1×: Tris base 3 g/L-Glycine 14.4 g/L). Blocking of non-specific binding was achieved by placing the membrane in a solution of non-fat dry milk (3%) overnight a 4 °C. Therefore, Bcl-2 antibody (1:1000, Sigma Aldrich) was incubated overnight at 4 °C; after washing with TBS-T solution (0.6% Trizma base; 0.87% NaCl; 0.05% Tween 20), the peroxidase-linked anti-mouse secondary antibody (0.04 µg/mL, Sigma Aldrich) was incubated for 2 h at room temperature. After other TBS-T washes, the presence of Bcl-2 protein was identify using “SuperSignal West Femto Maximum Sensitivity Substrate” (Thermo Scientific) and the chemiluminescence signal was reveal using the ChemiDoc XRS (Biorad, Hercules, CA, USA). β-actin (antibody anti-β-actin 0.4 µg/mL, Sigma Aldrich) was used to normalize each sample.

## Figures and Tables

**Table 1:** Weight average molecular weights of the polymer (MW) from Zimm plot analysis, mean hydrodynamic diameter (Dh) from DLS.

System	Dh (nm)		MW (MDa)	
	Mean	Width	Mean	Width
P*(0.1)AA50	15	6	0.65	0.07
P*(0.25)AA50	27	10	0.8	0.025
P*(0.25)AA25	31	10	0.78	0.056
P*(0.5)AA50	60	16	4.5	0.24

**Table 2:** Average carboxyl and amino groups per unit weigh of solid polymer (g) and per nanoparticle for the four NG variants. ζ-potential values from LDV.

System	COOH/NG	COOH/NG	NH_2_/NG	NH_2_/NG	ζ-Potential (mV)	
	mol/g	mol/mol	mol/g	mol/mol	Mean	Width
P*(0.1)AA50	1.1 × 10^−4^	71	5.4 × 10^−5^	35	−21.9	8.4
P*(0.25)AA50	1.5 × 10^−5^	19	2.1 × 10^−5^	24	−30.2	9.45
P*(0.25)AA25	3.6 × 10^−5^	28	1.8 × 10^−5^	15	−45.8	7.57
P*(0.5)AA50	2.3 × 10^−5^	67	2.9 × 10^−5^	83	−25.2	5.68

**Table 3:** Molar ratio between ligands and nanogels.

System	Conjugation Degree of AF	Conjugation Degree of Folic Acid
P*(0.1)AA50	4	15.5
P*(0.25)AA50	3.5	12
P*(0.25)AA25	1.5	4
P*(0.5)AA50	5	88
